# Generating Convergent Laguerre-Gaussian Beams Based on an Arrayed Convex Spiral Phaser Fabricated by 3D Printing

**DOI:** 10.3390/mi11080771

**Published:** 2020-08-13

**Authors:** Chang Liu, Chai Hu, Dong Wei, Mingce Chen, Jiashuo Shi, Haiwei Wang, Changsheng Xie, Xinyu Zhang

**Affiliations:** 1 National Key Laboratory of Science & Technology on Multispectral Information Processing, Huazhong University of Science & Technology, Wuhan 430074, China; M201772475@hust.edu.cn (C.L.); D201880681@hust.edu.cn (C.H.); D201677599@hust.edu.cn (D.W.); D201780651@hust.edu.cn (M.C.); D201980727@hust.edu.cn (J.S.); 2 School of Artificial Intelligence & Automation, Huazhong University of Science & Technology, Wuhan 430074, China; 3 Innovation Insititute, Huazhong University of Science & Technology, Wuhan 430074, China; 4 Wuhan National Laboratory for Optoelectronics, Huazhong University of Science & Technology, Wuhan 430074, China; hiway@hust.edu.cn (H.W.); Cs_xie@hust.edu.cn (C.X.)

**Keywords:** vortex beams, CSPA, 3D printing, two-photon absorption

## Abstract

A convex spiral phaser array (CSPA) is designed and fabricated to generate typical convergent Laguerre-Gaussian (LG) beams. A type of 3D printing technology based on the two-photon absorption effect is used to make the CSPAs with different featured sizes, which present a structural integrity and fabricating accuracy of ~200 nm according to the surface topography measurements. The light field vortex characteristics of the CSPAs are evaluated through illuminating them by lasers with different central wavelength such as 450 nm, 530 nm and 650 nm. It should be noted that the arrayed light fields out from the CSPA are all changed from a clockwise vortex orientation to a circular distribution at the focal plane and then a counterclockwise vortex orientation. The circular light field is distributed 380–400 μm away from the CSPA, which is close to the 370 μm of the focal plane design. The convergent LG beams can be effectively shaped by the CASPs produced.

## 1. Introduction

As known, vortex light beams demonstrate some potential applications such as quantum communication [1,2,3,4,5], trapping and manipulation of particles [6,7,8,9] and biomedicine [10,11,12], due to their unique orbital angular momentum [13,14,15,16,17]. In particular, the light fields of the Laguerre—Gaussian (LG) beams [18,19,20,21] contain the key Laguerre polynomials [22,23,24,25], which have a property of orthogonal normalization, so the LG beams can be used to form a complicated optical mode. Generally, any vortex beam can be considered as a linear combination of LG beams. Therefore, the research about LG beams becomes a hot topic and currently is mainly focused in the vortex beam characters. Considering the situation that the beam size will be widened gradually with the propagation distance, its application has still been greatly restricted.

A kind of convex spiral phaser array for generating convergent LG beams based on spiral phase plate (SPP) [26,27,28,29] is constructed by us. The fabrication of the micro-structures is completed using 3D printing technology [30,31,32,33]. According to the surface topography measurement charts, it can be seen that the micro-structures present an ideal manufacturing accuracy and a needed integrity. According to the measurements of their surface roughness, the processing accuracy is already about 200 nm. During the optical measurements, three lasers with different central wavelength such as 450 nm, 530 nm and 650 nm, are used. The light intensity distribution corresponding to different lasers has following characters in common: (1) the convergent LG beams being successfully generated and then converged at a distance of about 380–400 μm, which is already close to the focal plane expected at ~370 μm; (2) the light intensity distribution exhibiting a clockwise vortex orientation before the focal plane of CSPA and after the focal plane being changed to a counterclockwise vortex orientation, which means a vortex reversal corresponding to the focal plane of CSPA; (3) the light intensity being significantly low in the far field. The difference is that the number of the spiral lobes corresponding to different wavelength is variable but the same as the topological charge (TC) [34,35,36,37,38] corresponding to the light wavelength. It should be noted that the method of generation convergent LG beams achieved by us means a possibility for its efficient long-distance propagation.

## 2. Structure Design, Fabrication and Topography Measurement

### 2.1. Design and Fabrication

**Figure 1:**
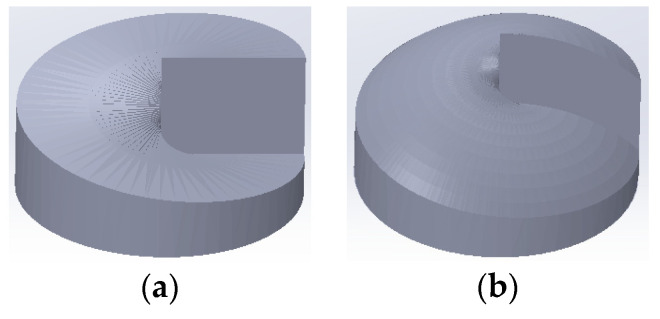
Structural diagram: (**a**) a SPP and (**b**) a convex spiral phaser.

In general, the method of combining a SPP and a convex lens can be used to present the effect of generation convergent LG beams. But this poorly integrated system will limit its application in imaging micro-systems, so as to inspire us to integrate a miniaturized convex lens and a SPP leading to a convex spiral phaser. A “Solidworks” software is utilized to draw a schematic diagram of the SPP and the convex spiral phaser, as shown in Figure 1. Comparing Figure 1a,b, it can be found that the top surface of the SPP is formed by a straight linear spiraling around a central-axis. The top surface of the convex spiral phaser is formed by a circular arc-spiraling around the central-axis. The ideal is to achieve a full beam convergence through this structure like a convex lens.

**Figure 2:**
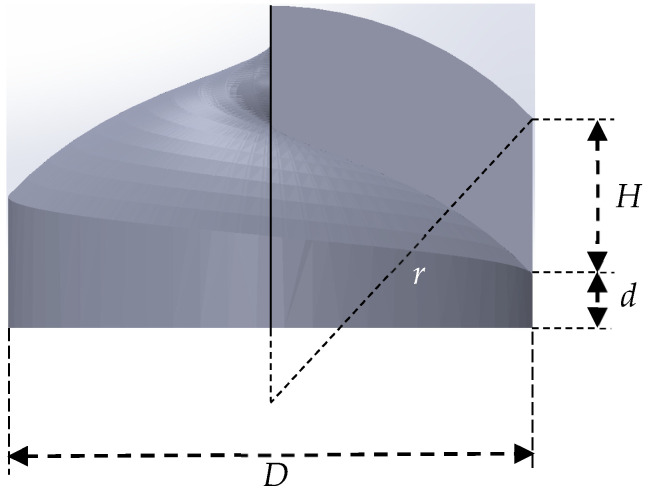
Parameter configuration: *r* being the curvature radius of the upper surface of the convex spiral phaser and *D* the diameter of the bottom circle and *H* the height of the cut surface and *d* the height of the base.

The main design parameters are shown in Figure 2. In formula (1), *n*_0_ represents the refractive index of the surrounding medium, and *n* the refractive index of the material for constructing the structure mentioned above, and *λ* the wavelength of incident light beams, and *l* the TC. Among them, *l* is generally an integer. If *H* is not an integer corresponding to the wavelength, the phase of incident beams at each phase step will be discontinuous, so as to destroy the circular intensity distribution of the transmitted light [39,40,41,42]. We set the central wavelength at ~650 nm, where the refractive index of Nanoscribe IP-Dip is 1.545 and the *l* being 5, and then the parameter *H* = 5.963 μm can be calculated. The value of the key parameters are shown in Table 1:

(1) $$l=\frac{H\left(n-n_{0}\right)}{\lambda }$$

From the parameter table, it can be seen that our processing accuracy is at the sub-micron level. In the past, due to the technological limitation, it is difficult to make a spiral phase plate with a relatively smooth surface, and generally a multi-level step spiral phase plate [43,44,45] is used instead. But with a rapid advancement of 3D printing technology, it becomes possible to shape a very smooth spiral phase plate through 3D manufacturing equipment according to the processing principle based on the two-photon absorption effect.

**Figure 3:**
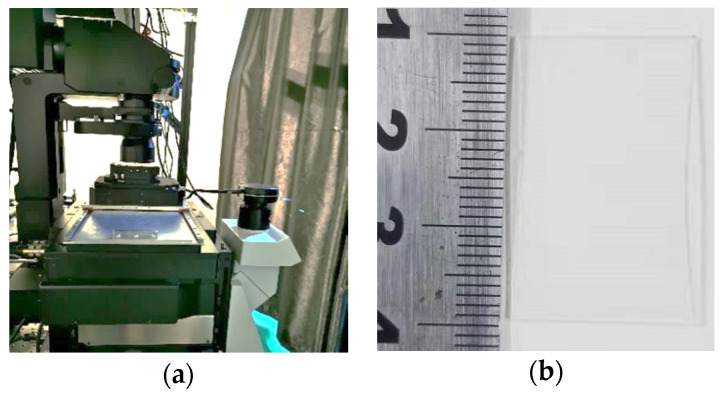
(**a**) Manufacturing equipment and (**b**) a sample fabricated.

In the actual processing, the photosensitive material of Nanoscribe IP-Dip is used, and the laser fabrication wavelength is ~780 nm, pulse width 120 fs, repetition frequency 80 MHz, peak power 6 kw, horizontal resolution ~200 nm, longitudinal resolution ~1 μm. First, the photosensitive material is spin-coated on a glass substrate, and then placed on a precision moving platform, and thus a focused laser beam is used to localized expose it. Generally, the two-photon absorption only occurs in a limited three-dimensional area near the focus of the 3D printer. So, the complete three-dimensional structure can be thoroughly exposed through moving the platform, and then the unexposed areas dissolved by acetone, and finally the residual solution air-dried with nitrogen. A 5 × 5 CSPA with a period of 25 μm and 30 μm is successfully fabricated, respectively. The manufacturing equipment (high-speed femtosecond laser three-dimensional direct writing system 1.0-HUST) and the fabricated sample are shown in Figure 3. From Figure 3b, we can see that because the material used for fabricating the sample is transparent in the visible wavelength region, the surface morphology of the sample cannot be directly observed. So, a laser confocal microscope is utilized to present the detailed structural character of the sample fabricated.

### 2.2. Topography Measurement

**Figure 4:**
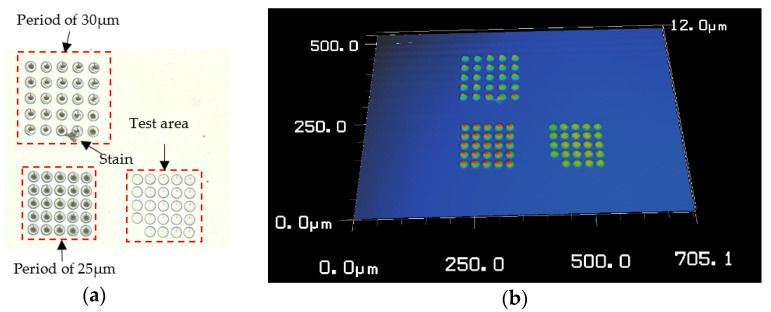
Measured overall morphology of the sample fabricated. (**a**) Two-dimensional topography, and (**b**) three-dimensional topography.

First, a VK-X200K laser confocal microscope (Japan Keyence Corporation) is utilized to perform the surface topography measurement. The overall morphology of the CSPA sample is shown in Figure 4. As shown, an initial test area is fabricated for performing equipment debugging before actually processing the CSPA samples. It can be seen that the overall morphology including both the samples with different period such as 25 μm and 30 μm and a test area with an unfilled small corner is roughly complete. The CSPA with a period of 30 μm has attached by a small stain nearing the bottom edge.

**Figure 5:**
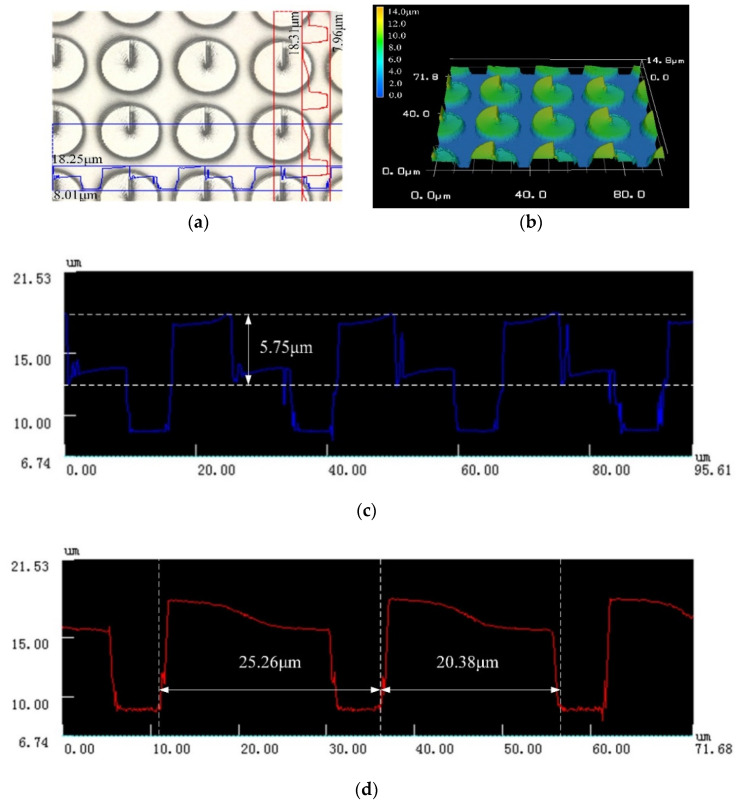
Appearance of a CSPA with a period of 25 μm. (**a**) Two-dimensional topography and (**b**) three-dimensional topography and both the surface profiles indicating the height or depth by (**c**) and the featured horizontal size by (**d**).

**Figure 6:**
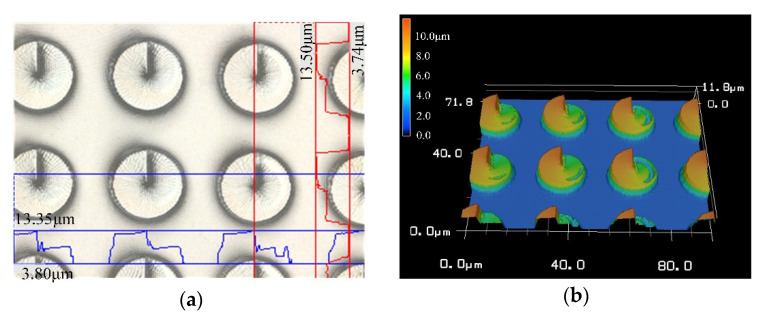

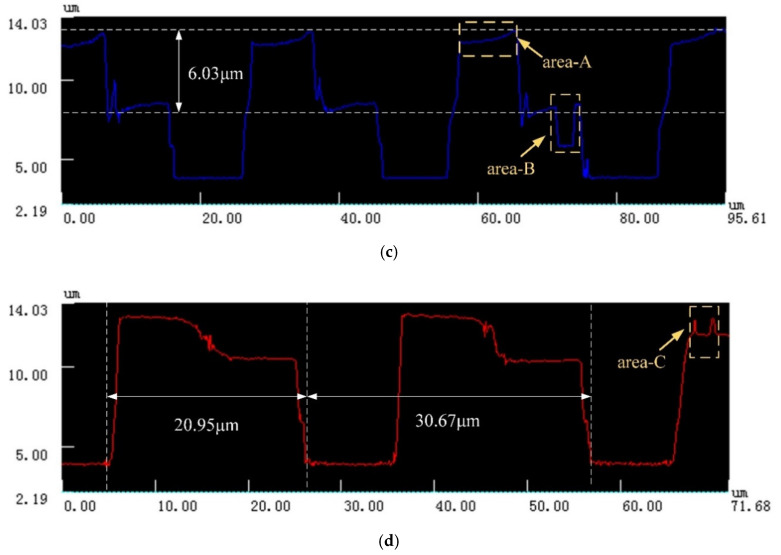
The CSPA with a 30 μm period. (**a**) Two-dimensional topography and (**b**) three-dimensional topography and both the surface profiles indicating the height or depth by (**c**) and the featured horizontal size by (**d**).

Next, a high-power objective lens is used to observe the details of the fabricated CSPAs. Figure 5 and Figure 6 are morphological diagrams of the CSPAs with a period of 25 μm and 30 μm, respectively. As shown in Figure 5, although existing some concave and convex burrs over small region of the CSPA with a period of 30 μm, the overall structure presents a better surface morphology and a high shaping precision of the phase step. According to the measurements, the structural error between the fabricated CSPA and the designed structure is within 5% to acquire an ideal fabrication result.

**Figure 7:**
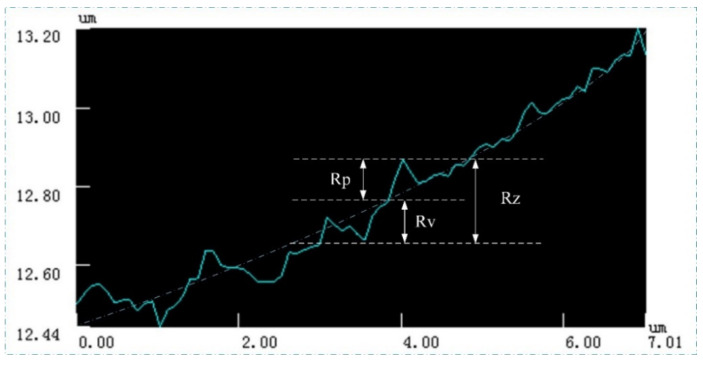
Enlarged view of the area-A.

**Figure 8:**
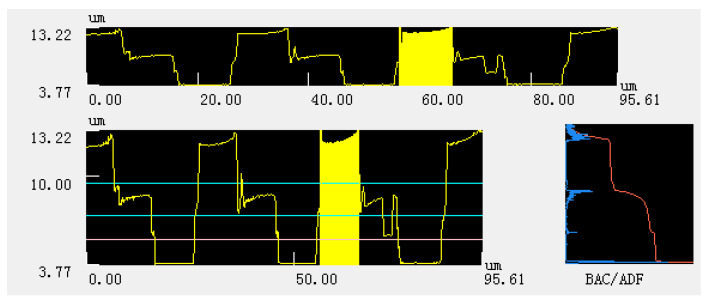
Quantitative analysis of surface roughness of area-A.

It can be seen from Figure 6 that the structural error of the acquired CSPA is still in a range of not exceeding 5%. However, several small concave and convex burrs on the surface of the structure indicated by areas B and C, which are more clear than that in Figure 5, can be observed. This is mainly because the photoinitiator molecules are excited by incident beams to generate free radicals and then undergo a chain reaction. The monomer molecules are polymerized to form high polymers. When the molecular weight in the polymer network reaches a critical value, the polymer will not be dissolved by subsequent immersion in acetone solution. The appearance of the concave area-B is due to the insufficient molecular weight of the polymer formed by the chain reaction, so as to lead to the subsequent dissolution when soaked in acetone, thereby forming a small pit. The convex area-C is due to the fact that the chain reaction occurs in a relatively large area, so as to result in the surrounding monomer molecules also forming a polymer. As shown, the height curve of the area-A shows an up and down fluctuation, which can be attributed to the machining accuracy limitation. Therefore, Figure 7 is an enlarged view of the overall contour of area-A. The roughness of area a is quantitatively detected with vk analysis software, as shown in Figure 8. The roughness parameters are shown in Table 2. As shown, the maximum roughness is approximately ~190 nm which is still less than the 3D printing accuracy of 200 nm. So, the surface of the manufactured sample is not smooth and thus presents a stepped profile, which is a typical diffraction phase outline.

## 3. Experimental Measurement

**Figure 9:**
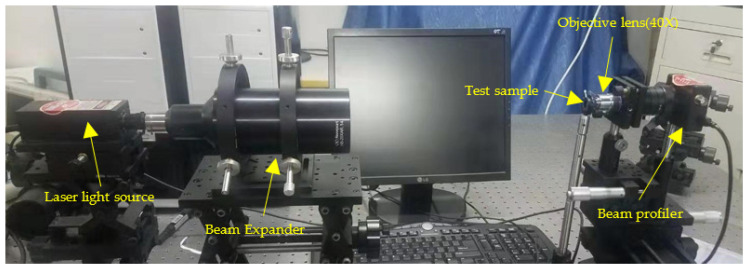
Measurement system for acquiring common optical characteristics of the CSPAs.

We set up a measurement system for acquiring common optical characteristics of the CSPAs. As shown in Figure 9, the laser beams firstly pass through a beam expander to form an uniform planewave, and then is incident upon the CSPA measured. After exiting from the CSPA, a vortex beam is formed. Finally, the shaped light intensity distribution is measured by a beam profiler. The detailed measuring operations are as follows.

**Figure 10:**
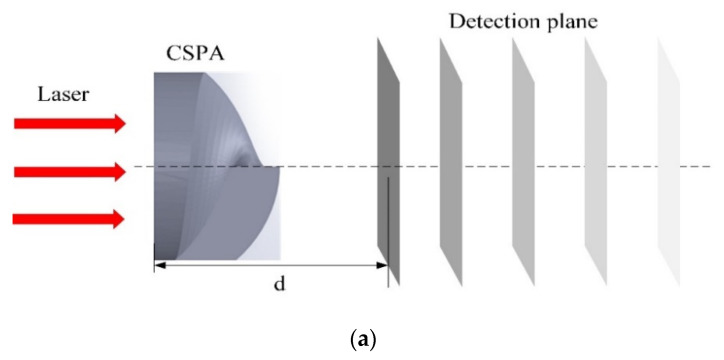

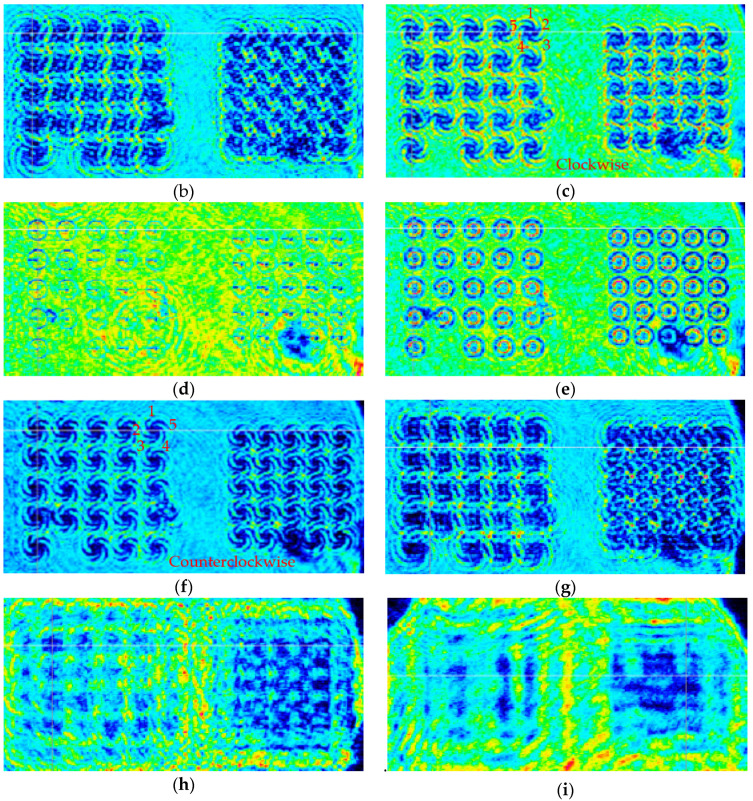
(**a**) Schematic diagram of the distance between CASP and the light field detection plane. The light intensity distribution of a center wavelength in the range of 635–671 nm laser beams, when the distance d is: (**b**) 200 μm, (**c**) 300 μm, (**d**) 380 μm, (**e**) 400 μm, (**f**) 500 μm, (**g**) 600 μm, (**h**) 3000 μm, and (**i**) 10,000 μm, respectively.

First, a red laser beam with a wavelength range of 635–671 nm is used to evaluate the CSPA fabricated. The light intensity distribution around the focal plane of the CSPA located at different distance are shown in Figure 10. It can be seen that the light field out of the CSPA initially exhibits a clockwise vortex distribution, and then the vortex-like light spot is gradually gathered as the distance increasing. When the distance is ~300 μm, the light intensity distribution presents the same morphology of the phaser. When the distance is further increased to ~400 μm, the light intensity distribution is already a micro-ring-shaped bright spot, which is a typical LG beam type of light field intensity distribution. When the distance continuously increasing, the exited light field becomes a counterclockwise vortex distribution, which is opposite to the initial vortex direction. Afterwards, the counterclockwise vortex distribution spread out gradually.

Then we amplify the field strength of the clockwise vortex distribution, the annular field intensity distribution, the counterclockwise vortex distribution, and the far field distribution. The obtained three-dimensional field intensity distribution map is obtained by subsequent measurement and does not correspond to the two-dimensional field intensity distribution map, and there are subtle differences. And according to Figure 10, it can be seen that the changes of the two periodic structures are basically the same, so we only intercept the three-dimensional field intensity distribution of the structure with a period of 25 μm.

**Figure 11:**
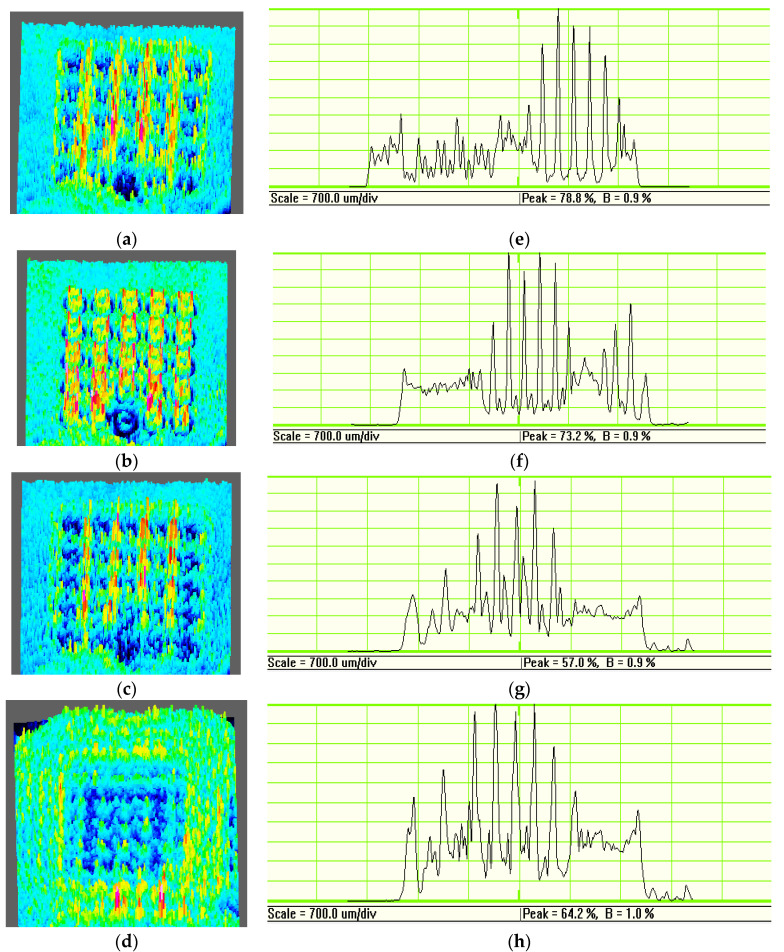
The three-dimension light intensity distribution of the laser beams with a center wavelength in the range of 635–671 nm, when the distance is: (**a**) 300 μm, (**b**) 400 μm, (**c**) 600 μm, and (**d**) 2500 μm, respectively. The scan and enlarged view of the annular LG beam at the focus, when the distance is: (**e**) 300 μm, (**f**) 400 μm, (**g**) 600 μm, and (**h**) 2500 μm, respectively.

From the Figure 11, we can see that before the focal plane, the three-dimensional distribution of the field strength in the area corresponding to the structure is concave, and the field strength is significantly lower than the surrounding area. The energy of the incident light field is mainly distributed in the blank areas between the structures. When the distance is increased to 400 μm, the field strength of the structure area is significantly higher than the field strength of the surrounding area. The incident field intensity distribution presents a bright ring shape, which is an obvious vortex beam field intensity distribution. When the distance continuously increasing, the ring-shaped bright spots gradually spread out, and the incident light field energy is gradually distributed in the blank areas between the structure. When the distance is increased to 2500 μm, the overall field strength of the array area is significantly lower than the surrounding area, and the incident light field energy distribution is the area outside the array structure. The radius of the bright ring is 10.75 μm through measurement. The scan and enlarged view of the annular LG beam at the focus is showed at Figure 11e–h. The noise level out of the ring structure can be obtained, the signal-to-noise ratios (SNRs) are 87.5 dB, 81.3 dB, 63.3 dB, 64.2 dB, respectively. The SNRs are all above 60 dB, and the signal parameters of the light field distribution diagram can effectively characterize the vortex light field of the plane.

**Figure 12:**
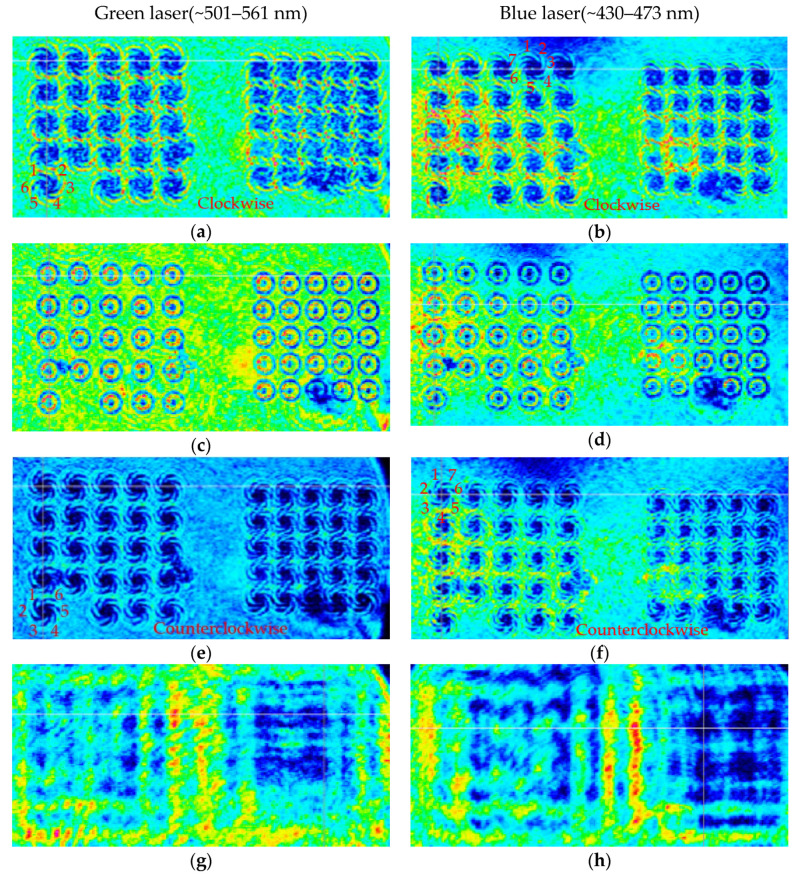
The light intensity distribution of the laser beams with a center wavelength in the range of 501–561 nm, when the distance is: (**a**) 300 μm, (**c**) 390 μm, (**e**) 500 μm, and (**g**) 10,000 μm, respectively. The light intensity distribution of the laser beams with a center wavelength in the range of 430–473 nm, when the distance is: (**b**) 300 μm, (**d**) 380 μm, (**f**) 500 μm, and (**h**) 10,000 μm, respectively.

Later, we replaced the red-light source with a green light source and a blue light source which with a center wavelength approximately in the range of 501–561 nm and 430–473 nm, respectively. The light intensity distributions at different distances are shown in Figure 12. It can be seen from Figure 12 that after passing through the convex spiral phaser, the field intensity distribution of both the green light beams and blue light beams is the same as that of red light beams. But light intensity distribution is changed from clockwise vortex to a circular distribution at the focal plane, and then continuously counterclockwise vortex. In the far field, the light intensity of the area corresponding to the CASP is also significantly lower. The number of the lobes of the vortex field is the same as the TC corresponding to its wavelength.

**Figure 13:**
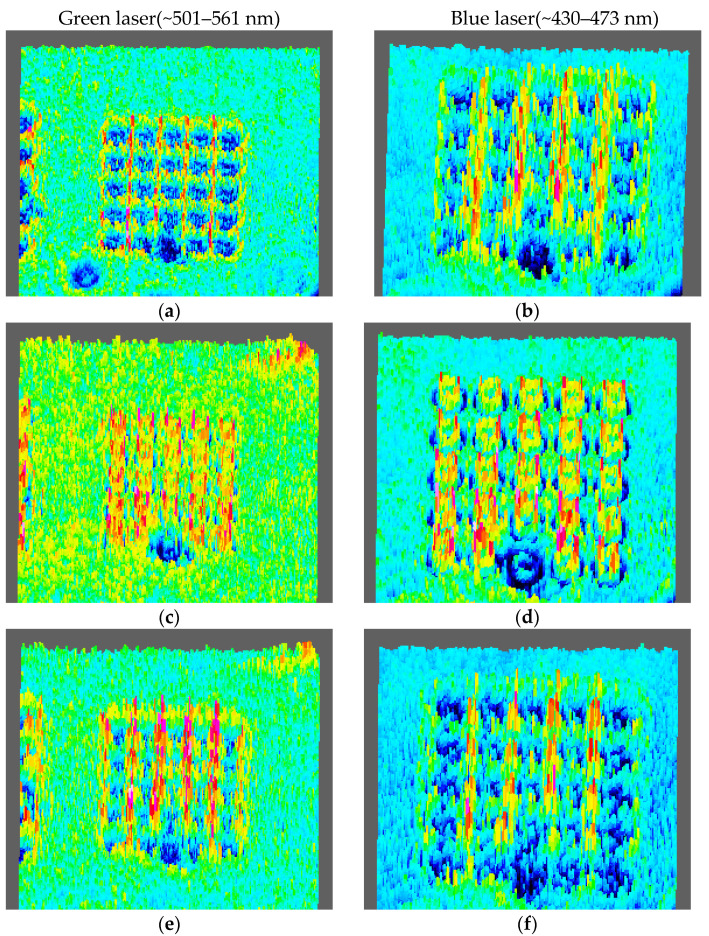

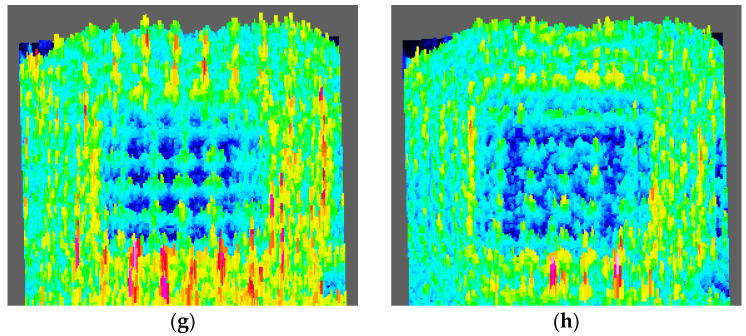
The three-dimension light intensity distribution of the laser beams with a center wavelength in the range of 501–561 nm, when the distance is: (**a**) 300 μm, (**c**) 390 μm, (**e**) 450 μm, and (**g**) 3000 μm, respectively. The light intensity distribution of the laser beam with a center wavelength in the range of 430–473 nm, when the distance is: (**b**) 300 μm, (**d**) 380 μm, (**f**) 440 μm, and (**h**) 3000 μm, respectively.

Similarly, from the Figure 13, we intercept the three-dimensional field intensity distribution of an array with a period of 25 μm under the illumination of a light source with a center wavelength of 530 nm and 450 nm, respectively. The change of the field intensity is the same. The incident light field energy in the area before and after the focal plane is mainly concentrated in the blank area between the structures. At the focal plane, the field intensity presents a bright ring distribution of a typical vortex beam and is located in the area of the structure. In the far field, the field strength of the array structure is significantly lower than the field strength of the surrounding un-structured area. The bright ring radius of the incident light with a central wavelength of 530 nm and 450 nm is 11.25 μm and 12.58 μm, respectively.

As shown, the light intensity of three light sources at the focal plane present in a ring shape, and the number of spiral lobes is also the same with the TC. However, neither the ring distribution nor the spiral lobe shape are ideal. This is mainly caused to the following reasons: The light source is broad-spectrum, not an ideal light source with a single wavelength. Therefore, light beams with different wavelengths are superimposed on the plane after passing through the CSAP, forming ghost noise; sidelobe noise comes from the interference between the central singular point and the edge of the aperture. In addition, the difference in intensity between the inside and outside of the aperture edge leads to straight-side diffraction, which intensifies the diffraction noise. 

## 4. Discussion

A 3D printing method is successfully used to fabricate CSPAs in this paper. Its theoretical processing accuracy is ~150 nm, and the actual processing accuracy ~200 nm according to practical measurements of the surface roughness. Due to the machining accuracy, the final stepped surface is not ideally smooth but already satisfy the requirement for generation a needed vortex light field. Considering the precision of focused ion beam (FIB) processing can reach an accuracy of ~4 nm, it can be expected that the CASPs will present a very smooth and entire surface through FIB technique. However, if the processing area being 150×150 μm or even larger and the etching depth being more than ~6 μm, the FIB processing will be time-consuming and expensive. It can be seen that the 3D printing technology has achieved a better balance between processing accuracy and fabrication cost for manufacturing large-area micro-optical devices.

It should be noted that from the light field characters and the light intensity distribution shown in Figure 9 and Figure 11, the secondary bright rings in addition to the main bright rings also exist at the focal plane. From the perspective of sidelobe suppression, the main bright rings are created by the diffraction of incident light beams passing through the outer circle area of the CSPA. The ineffective secondary bright rings or the sidelobe are caused by the diffraction of incident beam going through the central small region of each vortex phaser of the CSPA. Therefore, as long as an annular spiral phaser with an appropriate width being used, the operation of eliminating the sidelobe of the vortex beam should be achieved. In the follow-up work, we plan to hollow out the small central region of each vortex phaser of the CSPA in order to obtain the best annular light field.

Next we discuss the relationship between the bright ring radius and the wavelength of the incident light beam. In formula (2), *r* is the radius of the bright ring, and *l* the TC, and *w_0_* the beam waist radius of the Gaussian beam, and *z* the transmission distance, and *k* the wave number [46].

(2) $$r^{2}=\frac{\left|l\right|\left(k^{2}w_{0}^{4}+4z^{2}\right)}{2k^{2}w_{0}^{2}}$$

**Figure 14:**
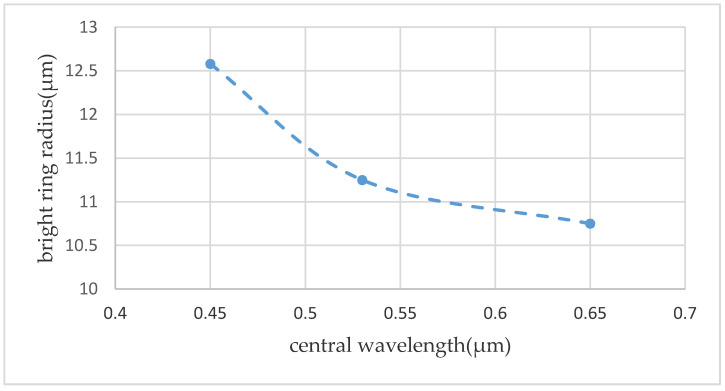
Relationship between the central wavelength and the radius of the bright ring.

Considering the safe optical power range that the detector can withstand and the convenience of matching the light source with the structural region, a beam expander is added behind the laser, so the planewave light source used in the experimental measurement. Since a planewave light being used, there is no corresponding parameter of the Gaussian beam, so it is impossible to draw an accurate relationship between the wavelength and the radius of the bright ring from the above formula. According to measurement results, the bright ring radius of beams including the red, the green, and the blue light, are obtained, which are 10.75 μm, 11.25 μm and 12.58 μm, respectively. We plot a relationship between the central wavelength of the incident light beams and the radius of the bright ring to obtain Figure 14. From the figure, we can see that as the wavelength increasing, the radius of the bright ring gradually decreased and then the decreasing trend tends to be gentle gradually. According to the following relation curve, the variation trend of the radius of the bright ring with the central wavelength of the incident beam is understood, which is convenient for predicting the bright ring radius corresponding to the incident beam with a wider wavelength range, and clarifies a good direction for in-depth exploration of the vortex optical field distribution.

## 5. Conclusions

In this paper, a new type of CSPA for generating convergent LG beams is proposed. A typical 3D printing technology is utilized to successfully produce CSAPs and then the processing accuracy already ~200 nm according to the measurements of the surface roughness. The fabricated CSPAs present a needed appearance. The common optical testing using three lasers with different central wavelength demonstrates that the obtained light fields are basically the same, and the light intensity distribution is effectively changed from a clockwise vortex to a focal circular light field distribution and then a counterclockwise vortex, which indicates an obvious vortex rotation redirection. In the far field, the vortex light field spreads gradually. It can be seen that the CSPA can successfully generate a convergent LG beams so as to lay a foundation for practical application of the LG beams in a long-distance range.

## Figures and Tables

**Table 1:** Parameter-Value correspondence table.

Parameter	Value (μm)
*r*	200
*R*	20
*d*	2
*H*	5.963

**Table 2:** Roughness related parameter table.

	Rp	Rv	Rz	Ra	Rq	Rsk	Rku	RΔq	RSm
Seg.1	0.09 μm	0.10 μm	0.19 μm	4.17 μm	4.17 μm	1.0002	1.0005	0.1922	0.00 μm
